# Micro-Nano formulation of bile-gut delivery: rheological, stability and cell survival, basal and maximum respiration studies

**DOI:** 10.1038/s41598-020-64355-z

**Published:** 2020-05-07

**Authors:** Susbin Raj Wagle, Daniel Walker, Bozica Kovacevic, Ahmed Gedawy, Momir Mikov, Svetlana Golocorbin-Kon, Armin Mooranian, Hani Al-Salami

**Affiliations:** 1 Biotechnology and Drug Development Research Laboratory, School of Pharmacy and Biomedical Sciences, Curtin Health Innovation Research Institute, Curtin University, Perth, Western Australia Australia; 2 Department of Pharmacology, Toxicology and Clinical Pharmacology, Faculty of Medicine, University of Novi Sad, Novi Sad, Serbia; 3 Department of Pharmacy, University of Novi Sad, Novi Sad, Serbia

**Keywords:** Nanoparticles, Drug delivery

## Abstract

Probucol (PB) is a drug that exhibits significant hydrophobicity and substantial intra and inter individual variability in oral absorption, with a miniature bioavailability and complex three compartmental pharmacokinetic modelling due to its high lipid affinity, low stability and high octanol to water partition coefficient. Multiple attempts to formulate PB have not produced satisfactory stable matrices, drug-release profile or rheological flow properties for optimum manufacturing conditions, and with positive and none toxic biological effects. Lithocholic acid (LCA) has recently shown to optimise formulation and cell uptake of drugs. Hence, the aim of this study was to design new PB delivery system, using LCA, and examine its morphology, rheology, stability, and cellular effects. PB was formulated with LCA and sodium alginate (PB-LCA-SA) using various microencapsulation methodologies, and best formulation was investigated *in vitro* and *ex vivo*. Using our Ionic Gelation Vibrational Jet flow technology, PB-LCA-SA microcapsules showed good stability and significantly enhanced cell viability, cellular respiration, and reduced inflammation suggesting potential LCA applications in PB delivery and biological effects.

## Introduction

More than 40% of drugs are lipophilic and exhibit poor water solubility and low bioavailability, despite favourable pharmacological activity^1^. A common and promising approach to improve the drug’s low water solubility and bioavailability is using encapsulation technology as well as formulation strategies to design new matrices where drugs can be incorporated, and delivered efficiently in the body, after oral administration^2–5^. Artificial cell microencapsulation (ACM) is widely used to microencapsulate and deliver lipophilic drugs that show poor dissolution and absorption kinetics and low bioavailability^6–8^. Microencapsulation of lipophilic drugs is one of the most promising applications in diabetes therapy and it encompasses the use of polymers/copolymers mixture to encapsulate and engulf a drug or a therapeutic entity conferring improved stability and targeted delivery properties^9–11^. The technology has been widely researched to optimise delivery and stability of therapeutics including drugs, viable cells and proteins^12–15^.

Diabetes Mellitus (DM) is a disease classified generally into two forms, type 1 diabetes (T1D) and type 2 diabetes (T2D)^16^. Millions of people are globally affected by DM and it is anticipated to reach more than 438 million by 2030, which is roughly 8% of the adult population^17,18^. Every region in the world has been affected by the disease, and its incidence is high in countries such as Australia, Europe, North America and countries undergoing westernization including India and China^19^. In 2016, it was estimated that total diabetes-related global costs more than 100 billion USD, which is more than 12% of global combined health expenditure^18,20^.

Insulin treatment is widely used in diabetes therapy, with all T1D patients, and in T2D more than one-third of T2D patients using insulin. Insulin is produced by pancreatic β-cells located in the islets of Langerhans. One of the common symptoms associated with T2D development and progression is chronic inflammation of β-cells as well as high levels of low-density lipoproteins (LDL), free radicals and oxidants, which have been connected to exacerbation and worsening of diabetes-associated complications^21^. Recent studies have shown that patients with lipid-disorders such as hypercholesterolemia are three times more likely to develop T2D and there is a significant association between T2D and cardiovascular diseases^22,23^. Published studies have also shown that significant inflammation and damage of pancreatic β-cell plays a major role in diabetes and cardiovascular disease development and progression, particularly since β-cells have limited defence against free radicals, oxidants and LDL-associated cellular toxicity^24,25^. Accordingly, new or adjunct antidiabetic drugs should exhibit anti-atherosclerotic, antilipidemic, antioxidant, anti-free radical and β-cell protective effects.

Probucol is a drug marketed for hypercholesteremia and remains widely prescribed in some countries such as China, and probably to a lesser extent in other countries such as Japan and India^26^. Despite its potent and powerful effects in lowering low density lipoprotein (LDL), probucol oral delivery has many challenges which resulted in its withdrawal from many countries including Australia and the USA. Probucol has high lipophilicity, poor water solubility, complex 3-compartmental pharmacokinetic modelling with variable and miniature bioavailability resulting in inconsistent oral uptake and severe side effects in some patients^26,27^. To make matters worse, probucol current oral dosage form, a tablet, has been used since its initial formulation development in the 1960s and remains under developed. Accordingly, in order to overcome challenges in probucol oral uptake in its current dosage form, new oral delivery matrices using cutting-edge technologies are needed. Such technologies can include microencapsulation with new excipients, that have demonstrated powerful oral targeted delivery, permeation enhancement properties and substantial consistent release profiles^6,28^.

Bile acids (BAs) are endogenously produced in animals and humans and notionally are known to facilitate food digestion, and vitamin absorption from the gastrointestinal tract. Due to their amphiphilic nature, recent studies have suggested potential applications of BAs in the oral delivery of lipophilic drugs, as formulation excipients, and permeation enhancers^29,30^. Common primary bile acids are cholic acid and chenodeoxycholic acid, while secondary and tertiary bile acids are deoxycholic acid, lithocholic acid, taurocholic acid, and ursodeoxycholic acid. Overall, there are more than 100 types of bile acids although their ratios in the gut-contents, blood, and tissues vary widely depending on species and health status^31^. Several studies in our lab, as well as others have attempted to design new and powerful nano and micro based delivery systems for probucol oral uptake, using some bile acids as well as other excipients’ combinations^8,10,32–34^. However, in preclinical studies, probucol absorption remains variable, and not ideal^30^. The bile acid lithocholic acid (LCA) has recently shown to optimise formulation and cell delivery^35^. It is one of the most prominent secondary bile acids and has recently displayed the potential application in the oral delivery of anticancer drugs^36^. It can self-assemble to develop nanostructure, and it has unique amphiphilic properties, high structural rigidity and reasonable biocompatibility^37^. LCA has anti-inflammatory properties and has shown positive effects on inflammatory bowel diseases^38^.

In previously published work, our lab demonstrated the potential application of bile acids, either alone or in combination with anti-diabetic drugs and polymers by forming microcapsules^8,10,39^. To date, ideal microcapsules for oral delivery of probucol would exhibit significant targeted properties, proper self-assemble profile, and unique amphiphilic properties with structural integrity and excellent biocompatibility, however this remains elusive and inconsistent. Thus, the objective of study is to examine the suitability, stability and biological activity of newly formed probucol microcapsules without and with lithocholic acid.

## Results and Discussion

### Morphology, size analysis and chemical characterization of microcapsules

**Figure 1:**
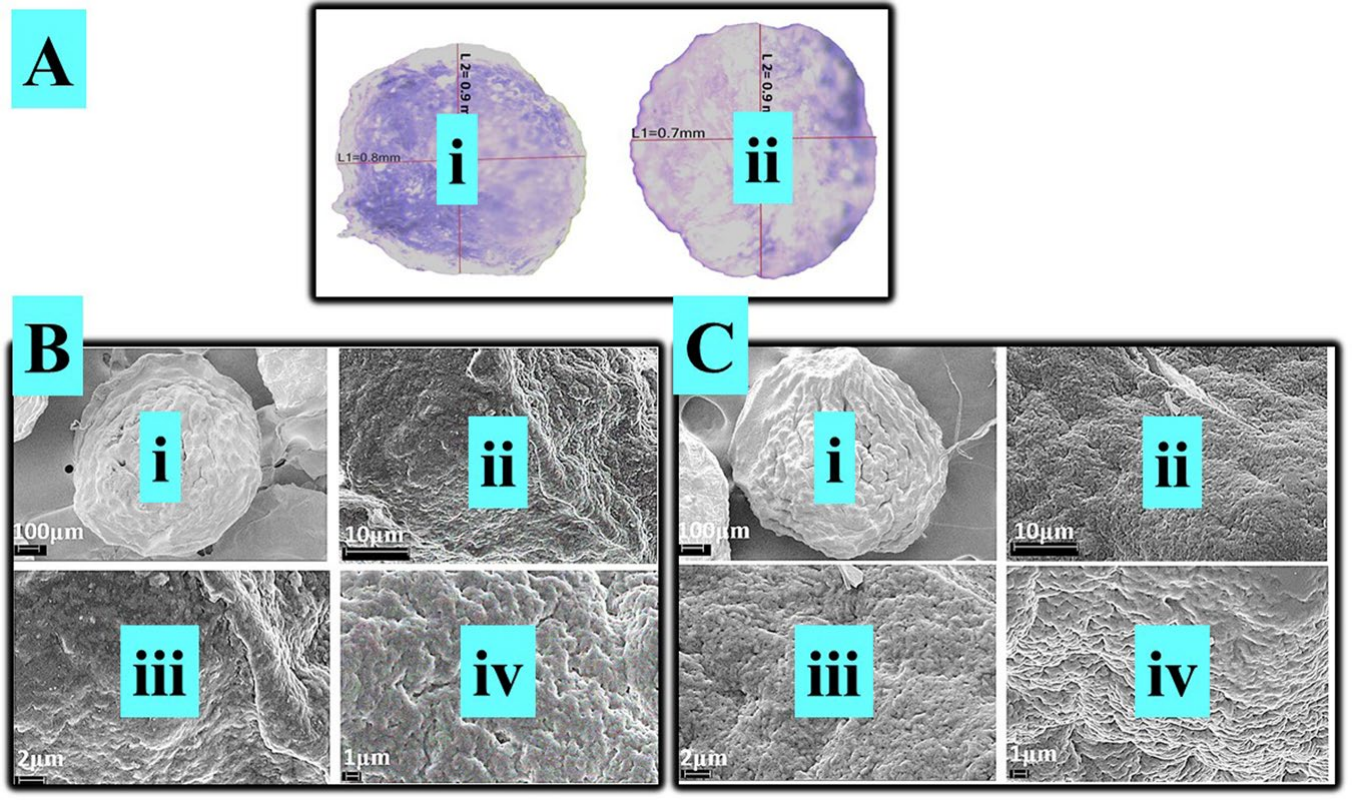
(**A**) Optical microscopy of PB-SA (i) and PB-LCA-SA (ii) microcapsules. (**B**)Scanning electron micrographs of PB-SA revealing microcapsules morphology and surface topography took at different magnifications, (i) 100 μm scale (ii) Surface morphology at 10 μm scale. (iii) 2 μm scale (iv) 1 μm scale. (**C**) Scanning electron micrographs of PB-LCA-SA and surface topography taken at different magnifications, (i) 100 μm scale (ii) Surface morphology at 10 μm scale. (iii) 2 μm scale (iv) 1 μm scale. PB- probucol; SA- sodium alginate; LCA-lithocholic acid.

**Figure 2:**
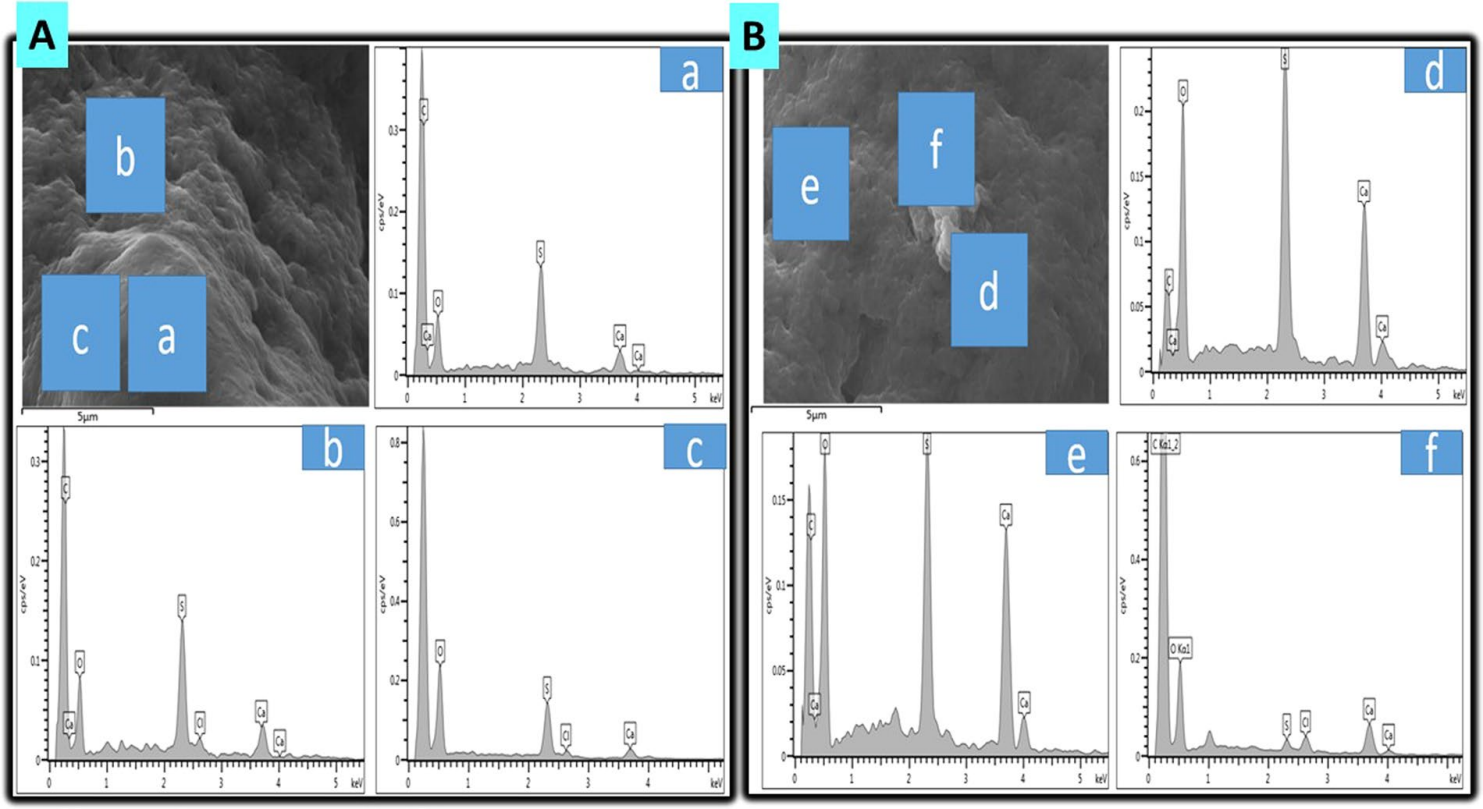
(**A**) EDS of PB-SA microcapsules and the corresponding elemental analysis (a-c). (**B**) EDS of PB-LCA-SA microcapsules and the corresponding elemental analysis (d-f). PB- probucol; SA- sodium alginate; LCA-lithocholic acid.

Figure 1(A) shows that optical microscopy (OM) examination of both formulations, PB-SA (i) and PB-LCA-SA (ii) maintained their spherical, round shape and similar size. OM results suggest that the incorporation of LCA did not significantly change microcapsules’ topography, uniformity, shape, size, or morphology. SEM (scanning electron microscope) results show well defined spherical shape microcapsule, Fig. 1B (i) and Fig. 1C (i). Microcapsules surface were rough, and Fig. 1B (ii), (iii), and (iv) and Fig. 1C (ii), (iii), and (iv) show that PB-SA and PB-LCA-SA microcapsules presented solid structure with small granules on the surface, which suggests that the incorporation of LCA into PB-SA formulation did not affect microcapsules morphology. Energy dispersive X-ray spectrometry (EDS) (Fig. 2) was used to analyse the element components present on the outer layer of the microcapsules. Figure 2A shows the surface analysis of PB-SA microcapsules and Fig. 2B shows the surface analysis of PB-LCA-SA microcapsules at different sites with their corresponding spectra. Three different locations were randomly chosen. These Figs. 2a–f represent the crystal deposition on microcapsules’ surface. Spectral analysis displays a high level of the sulphur atom in both formulations which indicate PB deposition^40^. Other elements such as O and C are anticipated to be present on the surface of microcapsules because these elements were part of the polymer and encapsulation processes deployed^41,42^. Figure 2 suggests that incorporation of LCA does not compromise or significantly alter the PB distribution within the microcapsules’ layers and potentially, PB release profile.

### Rheological, thermal and chemical profiles

**Figure 3:**
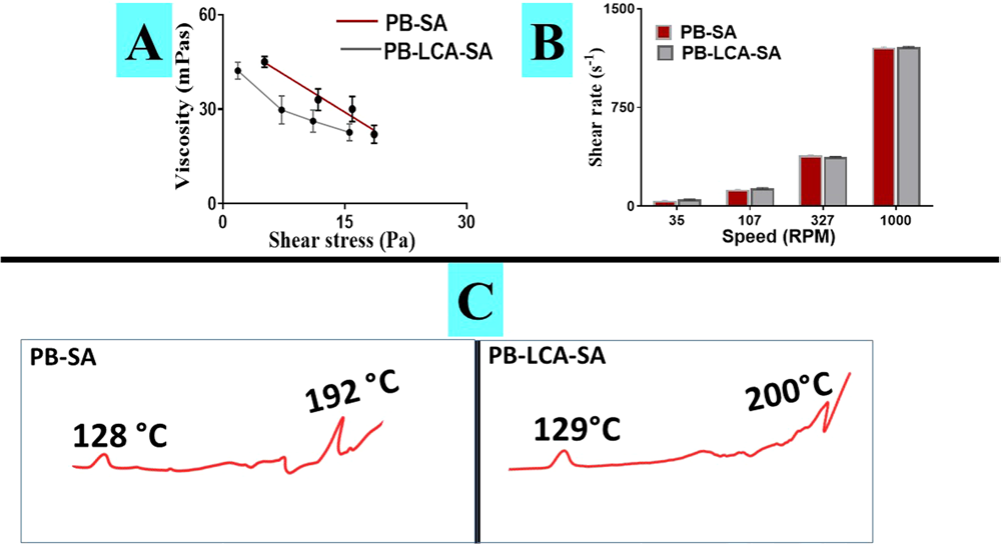
(**A**) Effect of shearing stress on viscosities, (**B**) Effect of speed on shear rate, (**C**) Dominant peaks observed by DSC analysis. PB- probucol; SA- sodium alginate; LCA-lithocholic acid.

For control and test formulations, Table 1 and Figs. 3A–B provide the rheological profiles, while Fig. 3C and Table 2 provide thermal and the chemical profiles.

**Table 1:** Rheological parameters for the formulations (n = 3, mean ± SEM, BLOD: Below Limit of Detection. PB- probucol; SA- sodium alginate; LCA-lithocholic acid.

Formula code	Set Speed	RPM	Torque(mNm)
PB-SA	2	35	0.1 ± 0.02
	4	107	0.35 ± 0.05
	6	327	0.8 ± 0.1
	8	1000	1.5 ± 0.03
PB-LCA-SA	2	35	BLOD
	4	107	0.14 ± 0.03
	6	327	0.36 ± 0.07
	8	1000	0.68 ± 0.17

**Table 2:** FTIR spectra of PB, SA and SA powder, PB-LCA-SA mixed powders and microcapsules. PB- probucol; SA- sodium alginate; LCA-lithocholic acid.

Formulation composition	FTIR spectra (λ cm^−1^)	Proposed functional groups
PB powder	2955.16, 1421 and 1308	O-H, C-H, S = O
SA powder	3253, 1569, 1405 and 1025	O-H, C = C, C-H, C-H
LCA powder	3282, 2925, 1701 and 1033	O-H, C-H, C = O, C-H
PB-LCA-SA powder	3270, 2926, 2854, 1697, 1600, 1308 and 1030	O-H, C-H, C-H, C = O, C = C, S = O, C-H
PB-SA microcapsules	3340, 2969, 1591, 1416, 1300 and 1026	O-H, C-H, C = C, C-H, S = O, C-H
PB-LCA-SA microcapsules	3320, 1593, 1712, 1417, 1300 and 1028	O-H, C = C, C = O, C-H, C-H, C-H

Torque profile was elevated by higher speed of mixing, and LCA incorporation did not change that, however, at highest speed, Torque remained lower when LCA was present, suggesting an alteration to the turning-power ability and resistance of the PB-SA formulation as a result of LCA incorporation. Similarly, both control and test formulations exhibited Non-Newtonian shear-thinning rheological behaviour with LCA incorporation resulting in sharper decrease in viscosity associated with consistent increase in shear stress. As mixing speed increases, shear rate of both formulation showed consistent increase, supporting a steady rheological profile with or without LCA. In addition, during mixing, both formulations form quick circular motions away from the site of centripetal force at an increasing speed of the stirring rod suggesting both formulations acted in a non-Weissenberg behaviour, which is consistent with previous studies^43,44^. These results provide useful new knowledge that can contribute to design of new formulation systems via demonstrating the effect of selective concentrations of LCA on the formulation rheology and force (and dimentions via Torque) needed for capsule fabrication and stability profiles.

The differential scanning calorimetry (DSC) thermal peaks provide information on thermal capacity and excipient-excipient and excipient-drug compatibilities. Thermal peak of PB powder is expected around 130 °C region, which correlates to PB melting temperature, while thermal peak of LCA powder is expected around 200 °C region, which correlates to LCA melting temperature. Thermal peak of SA powder is expected around 190 °C region, which correlates to SA melting temperature^45^. With the LCA and SA sharing similar melting temperatures, their peaks were expected to unit, which was observed in Fig. 3C, with potential amorphous phase formation. Results showed that the addition of LCA did not significantly alter the formulation heat capacity and did not result in significant changes of heat emission, or appearance of new peaks but slight endothermic peak shift^46^. Hence, during pre and post microencapsulation processes, the observed endothermic shift in the melting point of PB-SA, and PB-LCA-SA may be influenced by the ionic interaction between the ingredients constituting the microcapsules, alterations in the crystallinity, plasticization, and polymorphism of SA without compromising the overall stability^47^. Thermal compatibility of PB with other bile acids has been published^10^, supporting our finding. In our study, DSC analyses were complemented with FTIR (Fourier transform infrared**)** studies, to describe PB, LCA and SA stability and compatibility profiles.

FTIR studies are commonly used to examine the vibrational frequency levels of different functional groups present within the molecules or between molecules, which reflects chemical bond alteration and stability^48^. FTIR spectra were used to examine chemical stability and compatibility of PB with the polymer SA and the bile acid LCA pre and post-microencapsulation. PB, SA and LCA powders were analysed individually, combined, then pre and post encapsulation. The PB powder spectra analysis displayed three different characteristics peaks at 2955.16, 1421 and 1308 cm^−1^ which confirms the presence of its functional groups, and is in line with other studied (Table 2)^10,48,49^. Likewise, the SA powder analysis exhibited predominant O-H stretching intense peak at 3253 cm^−1^ and three medium intensity peaks at 1569, 1405 and 1025 cm^−1^ which is aligned with other studies^10,49^, while the spectra of LCA powder revealed distinct peaks at 3282, 2925, 1701 and 1033 cm^−1^ indicating the presence of LCA (Table 2). The combined mixtures PB-LCA-SA shows the distinct peaks of all compounds without any interference, alternations or dilution which suggests chemical compatibility pre-microencapsulation. PB-SA (control) microcapsules analysis reveals characteristic peaks at 1591, 1416, 1300 and 1026 cm^−1^ which correspond to PB and SA analysis (Table 2). Similar peaks were found after LCA addition (test) and the peak at 1712 cm^−1^ (C = O) confirms the presence of LCA. This result confirms an interaction between LCA and SA but not with PB as found in DSC results. A minor shift towards the right side of the PB peak was seen in both formulation that may have been brought out by SA or LCA. This interaction did not seem to affect PB peaks that remain present suggesting PB stability and excipient-compatibility (Table 2).

**Figure 4:**
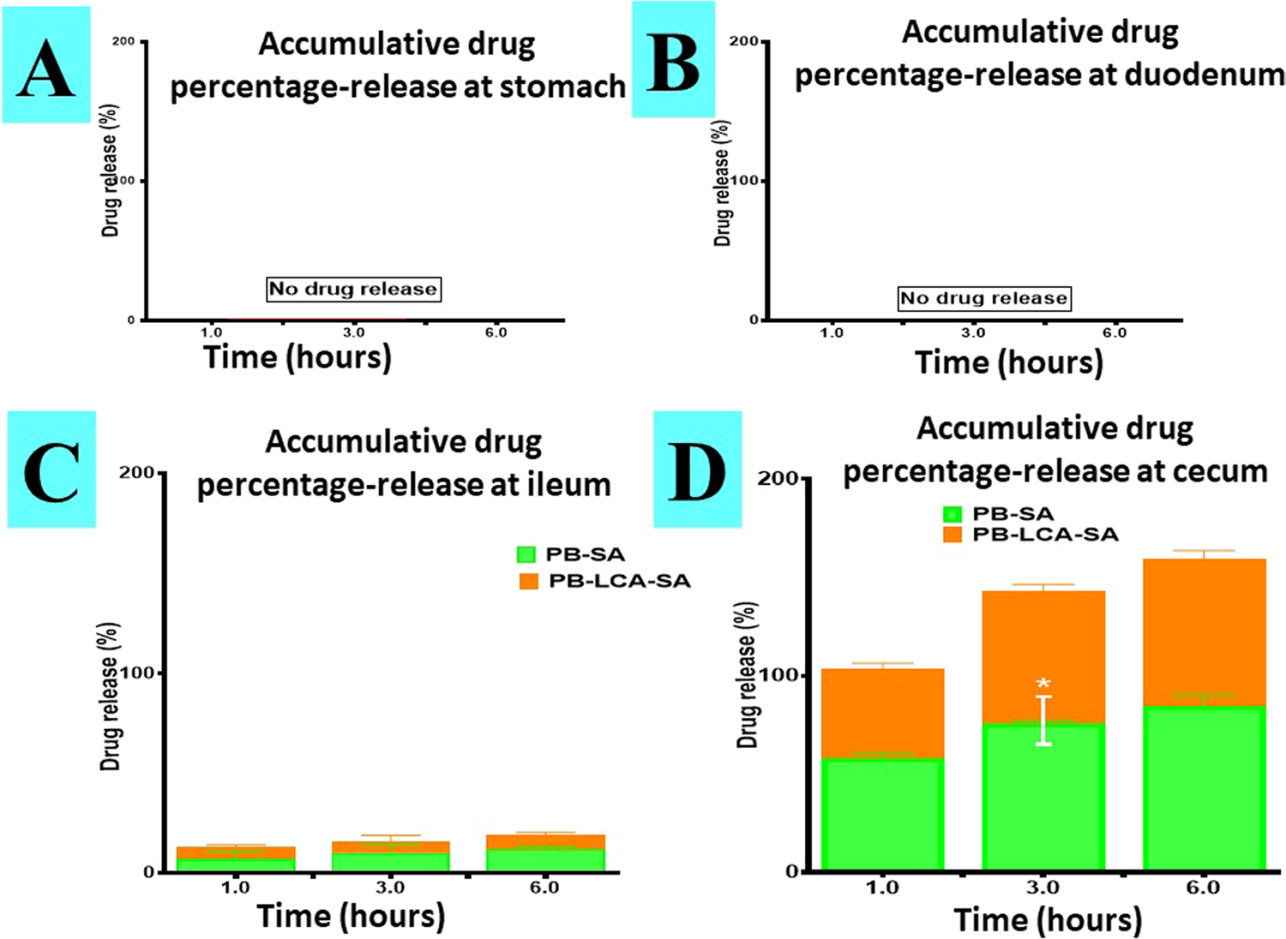
Microencapsules dissolution profiles in simulated gastric media at pH 1.5 (**A**), pH 3 (**B**), pH 6 (**C**) and pH 7.8 (**D**). N = 3, mean ± SD. PB- probucol; SA- sodium alginate; LCA-lithocholic acid.*p < 0.05.

LCA incorporation into PB microcapsules did not seem to affect compatibility or stability profiles of the microcapsules, but may influence PB release profiles from the microcapsules (Fig. 4).

### Microcapsule disintegration, drug release and dissolution studies

Figure 4 shows PB release from control (PB-SA) and test (PB-LCA-SA) microcapsules in gut-simulating media, at four pH (1.5, 3, 6 and 7.8) over 6 hours at 37 °C. The four (Fig. 4A–D) conditions represent four different sites of PB absorption in the gastrointestinal tract^10,50,51^. PB microcapsules demonstrated significant dependence on the gut media rather than LCA incorporation. At lower pH (stomach & duodenum), there was negligible release from control and test microcapsules, while at higher pH (ileum) there was small drug release, with no significant effects from LCA incorporation. At high pH (cecum) there was substantial PB release with more than 50% within the first hour (Fig. 4D). The incorporation of LCA reduced PB release after 3 hours (p < 0.05) and the overall release profiles of control and test remain similar at the end of the 6 hours experiment suggesting possibly a slightly more controlled PB release by LCA incorporation. At low pH (1.5, 3 and 6) alginic acid present on SA matrix results in shrinkage of alginate and thus encapsulated PB remained within core of the microcapsules but in higher pH (> 6) due to rapid dissolution and solubilisation, alginic acid forms a soluble viscous layer^52^, resulting in the burst of PB. Similar studies have shown various and inconsistent effects of bile acids on drug release profiles, suggesting drug release from bile acid microcapsules can be formulation dependent^10,51^.

### Biological activity of PB-loaded microcapsules

**Figure 5:**
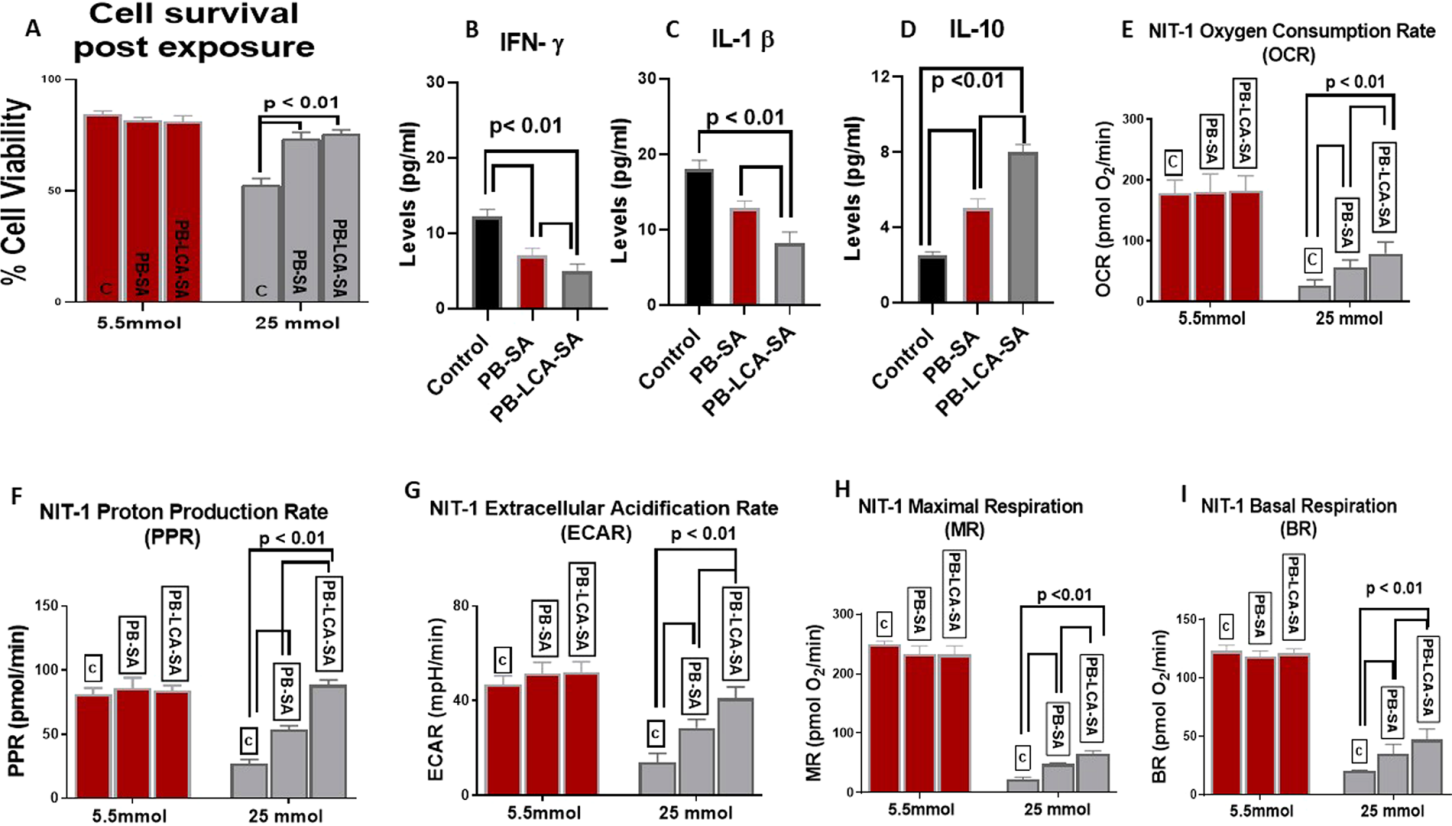
NIT-1 cell viability at 5.5 mmol and 25 mmol glucose (**A**), (**B**–**D**) NIT-1 level of inflammatory cytokines production, (**E**–**I**) bioenergetics parameters for NIT-1 beta cells at 5.5 mmol and 25 mmol glucose treated with PB-SA and PB-LCA-SA microcapsules. PB- probucol; SA- sodium alginate; LCA-lithocholic acid. Data values are mean ± SD, N = 3; p < 0.01.

Figure 5 shows cell viability at normoglycaemic (5.5 mmol) and hyperglycaemic (25 mmol) states (Fig. 5A), inflammatory (Fig. 5B-D) and bioenergetics (Fig. 5E-I) biological profiles of pancreatic β-cells line exposed for 48 hours to negative control (c; empty microcapsules), PB-SA and PB-LCA-SA microcapsules.

Figure 5A shows that at the hyperglycaemic state, PB-LCA-SA showed significantly higher cell viability compared with PB-SA which was higher than the sham control group, while at the normoglycaemic state, cell viability remained similar regardless of treatments. The higher cell viability, at the hyperglycaemic state as the result of treatments, suggests cellular protective effects brought about by PB, and enhanced by LCA, when encapsulated. In the literature, PB exhibits potent anti-oxidant, anti-inflammatory, and β cells protection properties, and has shown to ameliorate hyperglycaemia-induced oxidative stress and inflammation^53,54^. Results suggest that LCA incorporation did not compromise cell viability, which might be due to modulation of the inflammatory and bioenergetics profiles. Figure 5(B–D) show that PB-SA exerted an anti-inflammatory effects via reducing levels of proinflammatory cytokines (IFN-γ and IL-1 β) and enhancing levels of anti-inflammatory cytokine (IL-10), which is consistent with findings from Fig. 5A, and may relate directly to the controlled-release effects of LCA observed in Fig. 4D. Figure 5(E-I) show mitochondrial activity and cellular respiration of pancreatic β-cells at the normoglycaemic and hyperglycaemic states and results are in line with cell viability and inflammatory profiles and demonstrate significant activation of oxygen consumption rate (OCR), extracellular acidification rate (ECAR), proton production rate (PPR), maximal respiration (MR) and basal respiration (BR) values when cells were exposed to PB-SA and PB-LCA-SA microcapsules compare with the hyperglycaemic control. Findings suggest that PB exerted a pancreatic β-cell protective effects possibly via reducing inflammation and enhancing biological bioenergetics, and these effects were optimised by LCA incorporation into PB microcapsules. At the normoglyacemic state, cellular bioenergetics remained similar regardless of treatments, while at the hyperglycaemic state, the treatments showed significant positive effects on biomarkers of cellular metabolism (PPR) and energetics (BR and MR) which were significantly and negatively affected at the hyperglycaemic state. For cellular respiration and acidification rates (OCR and ECAR), both treatments (PB-SA and PB-LCA-SA) showed consistent improvements to cellular bioenergetics and optimised biological functions at the hyperglycaemic states, which supports cellular biological improvements and viability results (Fig. 5). It is worth stating that significant elevation of bioenergetics biomarkers observed when cell treated with both microcapsules at the hyperglycaemic state, suggests that there is increased mitochondrial respiration and higher oxygen molecules serving as electron acceptors and assisting activity within the electron transport chain, with a proportional increase in oxidative phosphorylation, which is consistent with our previous studies^55,56^.

Biological measurements (Fig. 5) have shown positive and significant cell-protective, anti-inflammatory and pro-bioenergetics effects of the encapsulated PB and PB-LCA microcapsules on pancreatic β-cells. Compared with the literature, LCA on PB cellular effects are consistent with published data that have shown that bile acids as well as PB-loaded microcapsules enhanced beta-cell viability and functions^8,57^. In one of our recent studies, the bile acid ursodeoxycholic acid was incorporated with drug-containing microcapsules, and showed similar effects on drug release, and cell viability, and these effects were formulation-dependent^31^. Overall, this is the first study to elucidate LCA applications in PB-microcapsules’, in the context of cell protection and diabetes treatment.

**Figure 6:**
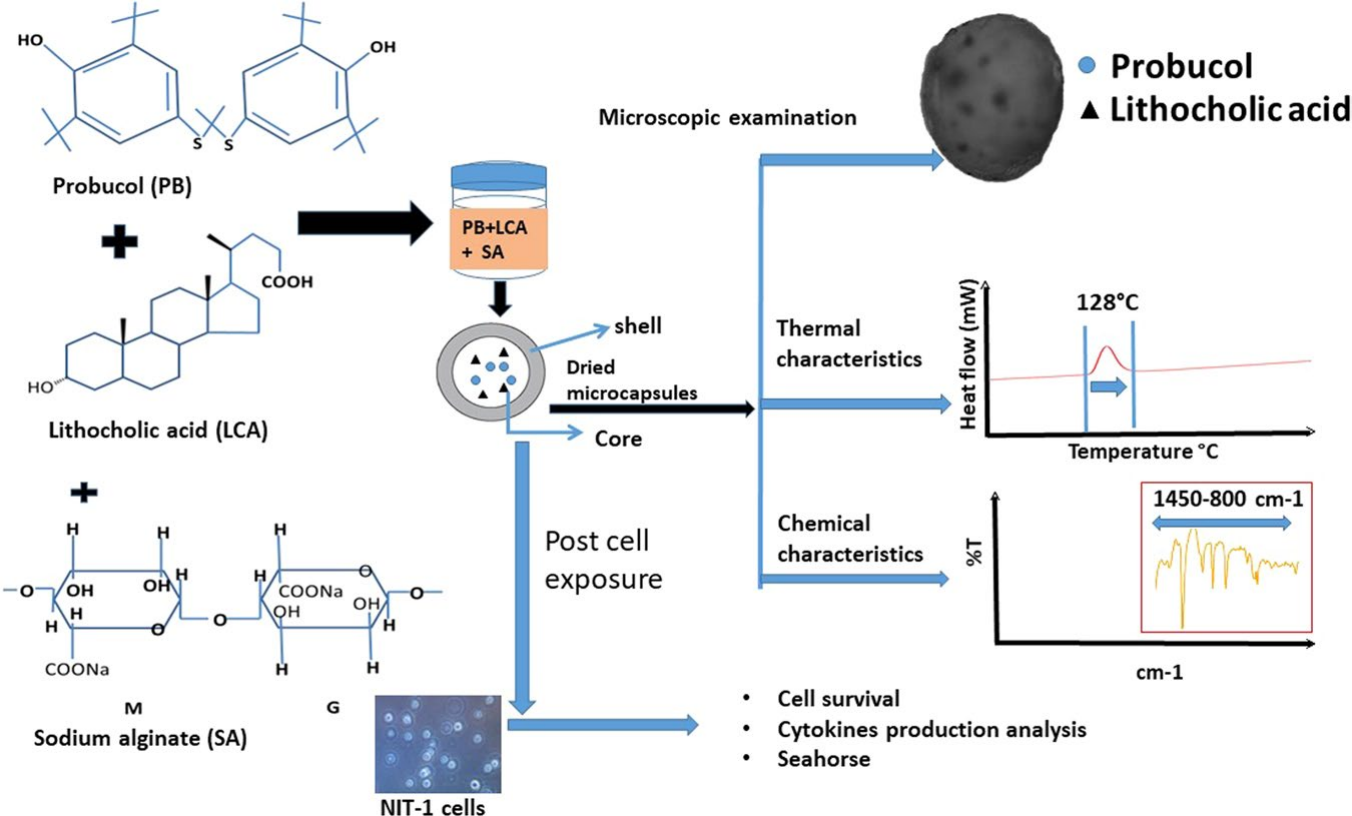
The summary figure of the study.

In conclusion, this study investigated the *in vitro* and *ex vivo* effects of LCA on PB-SA microcapsules in terms of capsules’ morphology, rheological, thermal and chemical profiles, and biological effects on pancreatic β-cells. Results showed that the LCA did not compromise the pharmaceutical quality of the PB microcapsules, and it enhanced PB release profile as well as the biological effects at the hyperglycaemic state suggesting potential beneficial effects in diabetes therapy. The overall procedure along with the study results is presented in Fig. 6.

## Materials and Method

### Materials

Probucol (PB; 99.89%) and sodium alginate (SA; 99%) were obtained from Sigma-Aldrich, (St Louis, MO, USA) respectively. Lithocholic acid (LCA; ≥ 95%) was purchased from Sigma- Aldrich, Co., USA. Calcium chloride dehydrate (98%) was purchased from Scharlab S.L (Australia). All other required chemical and solvents were obtained from Merck and Co, and were of analytical grade and used without any purification.

### Cell lines

The mouse pancreatic β cells line NIT-1 was gifted from Prof Morahan (The University of Western Australia).

### Drug preparations

Drug stock suspension of PB (2.5 mg/ml) and LCA (1 mg/ml) were made by adding powder with 5% ultra-water-soluble gel. The 2% w/v CaCl_2_ standard solution was prepared by mixing CaCl_2_ with deionized water. All chemical and solvents were thoroughly mixed for 10 hours at room temperature, kept in the fridge and processed within 48 hours of preparation.

### Microcapsules production

PB-SA and PB-LCA-SA loaded low viscosity SA microcapsule were prepared by using ionic gelation vibrational jet flow technique (BUCHI Labortechnik, Switzerland), in a constant ratio of 1:30 and 1:3:30 respectively. This proportion was based upon the previous publications^9,10^. The parameters were fixed in a frequency range of 1500-2000 Hz with a constant air pressure of 350 mbar with a flow rate of 5 ml/minute. All formulations and microcapsules (PB-SA and PB-LCA-SA) were made, and three independent lots of microcapsules were produced (n = 3).

### Characterization of loaded microcapsules

#### Morphology, size analysis and chemical characterization of microcapsules

All microcapsules (both formulations) were prepared and analysed within 48 hours. The morphological analysis and size of microcapsules were undertaken by using OM, SEM and EDS. The microcapsules’ diameters were calculated with the help of the software ToupTek which is provided within the instrument.

Briefly, 20 freshly prepared microcapsules were dried and randomly taken to access the morphological characteristics and microcapsules’ diameter using an optical microscope (Nikon SM2800, Japan mounted with Toup-view Photonics, Co., Ltd Hangzhou, China).

SEM (Neon 40EsB FIB-SEM; Zeiss, Oberkochen, Germany) was used to measure the surface morphology of microcapsules. Multiple pictures from different angles and multiple scales were performed to capture the details of the surface topography. To interpret atoms distribution present in microcapsules, was obtained by using EDS (INCA X-Act; Oxford Instruments, UK). Before analysis, dried microcapsules were mounted on a glass stub and coated under vacuum^10^.

#### Determination of rheological parameters

Rheological parameters including viscosity, shear stress and torque of both formulations were done for freshly made mixtures (prior to gelation), using 2 ml aliquots (n = 3) at room temperature (Visco-88 viscometer, Malvern Instruments, Malvern, UK).

#### Thermal analysis

Thermal analysis was undertaken by DSC (DSC 8000, PerkinElmer Inc., Waltham, MA, USA). Five mg of PB powder or its microcapsules were loaded into a sealed pan and heated at 30 °C per minute at a flow rate of 20 ml/minutes under nitrogen in the 30- 250 range. For reference (control), an empty aluminium pan was utilised.

#### Chemical stability studies

FTIR was deployed to determine the chemistry profiles of each formulation and the prepared microcapsules. The spectra of drug, their physical mixtures and formulated microcapsules were measured by FTIR spectrometer-TWO (PerkinElmer Inc., Waltham, MA, USA) in transmission in the frequency range 450-4000 cm^−1^.

### Microcapsule disintegration, drug release and dissolution measurements

Two and a half grams of microcapsules were weighed and suspended in 100 ml of simulated intestinal fluids at four different pH values of 1.5, 3, 6 and 7.8 at 37 °C. The sink condition was maintained throughout the experiment, and the dissolution medium was stirred at 200 rpm for 6hrs^10^. PB concentrations were measured with a UV spectrophotometer (Schimadzu UV-Vis spectrophotometer 1240, Japan) at 242 nm using our published methods^10^. To exclude any interferences and ensure only PB was being measured at this particular wavelength, microcapsules without drug (SA microcapsules) were also analysed in all four pH values. The study was carried out in triplicate (n = 3).

#### Pancreatic NIT-1 β cells biological examination

The cells were stored in liquid nitrogen and were cultured on T-75 cm^2^ flasks (Thermo Fisher Scientific, Australia) with Dulbecco’s Eagle Medium (DMEM) (Sigma-Aldrich, USA) supplemented with 10% foetal bovine serum (Thermo Fisher Scientific, Australia), 5.5 mmol glucose (Sigma-Aldrich, USA) and 1% penicillin streptomycin (Gibco, Life Technologies, USA)^58^.

The MTT assays (3- (4, 5 - dimethylthiazol-2-yl)-2, 5- diphenyltetrazolium bromide) was used to determine cellular viability of NIT-1 cells after exposure with microcapsules at two glucose concentration (5.5 mmol and 25 mmol) over 48 hours. The MTT stock solution (5 mg/ml) (Sigma Chemical CO, USA) was prepared using phosphate buffer at pH 7.4 (Thermo Fisher Scientific, Australia). A well-established method was used to determine the cellular viability of microcapsules treated NIT-1 cells^8,58,59^. Briefly, after 48 hours of incubation, microcapsules were removed from the 96 wells plates (Thermo Fisher Scientific, Australia) that have been placed in 200 µl of media (pH 7.4) and 20 µl of MTT from the prepared stock solution were added into each 96-wells plates. After 4 hours, MTT conversion to formazan was stopped by adding DMSO (Sigma Chemical CO, USA). The MTT assay was performed by using microplate spectrophotometer system (PerkinElmer Multimode Plate Readers, USA) at 550 nm.

The evaluation of mitochondrial activities of microcapsules treated NIT-1 cells were done in real-time using an in-house developed method with Seahorse Flux Analyser XF 96 (Seahorse Bioscience, USA)^8^.

The level of cytokines (pro-inflammatory and anti-inflammatory) production were measured to test the effect of PB loaded microcapsules on treated NIT-1 cells. NIT-1 cells were cultured in DMEM medium with microcapsules at glucose concentration of 5.5 mmol and 25 mmol for 48 hours and microcapsules were removed and aliquots of the media were tested for IL-10, IL-1 beta, and IFN- gamma via cytokine bead array flow cytometric analysis (BD Bioscience cytometric Bead Array Mouse, USA)^8,10^ using cell analyzer BD FACSCanto II (BD Bioscience, USA). Data analysis was carried out using the computer software FlowJO (FlowJo, Ashland, Oregon).

### Statistical analysis

Graph Pad Prism version X8.2 (Graphpad, Inc., USA) was used to create graph and results are presented as mean ± SD. Statistical measurements were carried out using parametric/non-parametric analysis or using a one way ANOVA and a Tuckey post-hoc, as appropriate set the level of significance.
